# A Vaccine Targeted at CETP Alleviates High Fat and High Cholesterol Diet-Induced Atherosclerosis and Non-Alcoholic Steatohepatitis in Rabbit

**DOI:** 10.1371/journal.pone.0111529

**Published:** 2014-12-08

**Authors:** Yi-Wei Liaw, Chi-Yu Lin, Yu-Sheng Lai, Tzu-Chung Yang, Chau-Jong Wang, Jacqueline Whang-Peng, Leroy F. Liu, Chia-Po Lin, Shin Nieh, Shao-Chun Lu, Jaulang Hwang

**Affiliations:** 1 Institute of Biochemistry and Molecular Biology, National Yang-Ming University, Taipei, Taiwan; 2 Department of Biochemistry and Molecular Biology, College of Medicine, National Taiwan University, Taipei, Taiwan; 3 Graduate Institute of Life Sciences, National Defense Medical Center, Taipei, Taiwan; 4 Institute of Biochemistry and Biotechnology, Chung Shan Medical University, Taichung, Taiwan; 5 Institute of Cancer Biology and Drug Discovery, Taipei Medical University, Taipei, Taiwan; 6 Department of Pharmacology, The University of Medicine and Dentistry of New Jersey, Robert Wood Johnson Medical School, Piscataway, New Jersey, United States of America; 7 Division of Drug Biology, Bureau of Food and Drug Analysis, Department of Health, Executive Yuan, Taiwan; 8 Department of Pathology, National Defense Medical Center and Tri-Service General Hospital, Taipei, Taiwan; 9 Department of Biochemistry, Medical College, Taipei Medical University, Taipei, Taiwan; University of Basque Country, Spain

## Abstract

Low HDL-C levels are associated with atherosclerosis and non-alcoholic steatohepatitis, and increased levels may reduce the risk of these diseases. Inhibition of cholesteryl ester transfer protein (CETP) activity is considered a promising strategy for increasing HDL-C levels. Since CETP is a self-antigen with low immunogenicity, we developed a novel CETP vaccine (Fc-CETP_6_) to overcome the low immunogenicity of CETP and for long-term inhibition of CETP activity. The vaccine consists of a rabbit IgG Fc domain for antigen delivery to antigen-presenting cells fused to a linear array of 6 repeats of a CETP epitope to efficiently activate B cells. Rabbits were fed a high fat/cholesterol (HFC) diet to induce atherosclerosis and NASH, and immunized with Fc-CETP_6_ vaccine. The Fc-CETP_6_ vaccine successfully elicited anti-CETP antibodies and lowered plasma CETP activity. The levels of plasma HDL-C and ApoA-I were higher, and plasma ox-LDL lower, in the Fc-CETP_6_-immunized rabbits as compared to the unimmunized HFC diet-fed rabbits. Pathological analyses revealed less lipid accumulation and inflammation in the aorta and liver of the Fc-CETP_6_-immunized rabbits. These results show that the Fc-CETP_6_ vaccine efficiently elicited antibodies against CETP and reduced susceptibility to both atherosclerosis and steatohepatitis induced by the HFC diet. Our findings suggest that the Fc-CETP_6_ vaccine may improve atherosclerosis and NASH and has high potential for clinical use.

## Introduction

HDL continues to attract interest because its levels are inversely associated with the risk of cardiovascular disease [1]. This may be attributed to its having various potentially anti-atherogenic properties, such as reverse cholesterol transport, anti-inflammatory, anti-oxidative, and anti-thrombotic effects [2]. Clinical studies have shown that low HDL-C is also found in non-alcoholic steatohepatitis (NASH) [3]. In addition, patients with hepatic steatosis also showed higher CETP activity [4]. NASH shares several characteristics with atherosclerosis, including lipid accumulation, inflammation, and macrophage infiltration [5]. Recent studies have suggested that the pathogenesis of NASH involves scavenger receptor-mediated uptake of ox-LDL by macrophages in the liver [6], [7]. This may explain, at least in part, why NASH is an important risk factor for cardiovascular disease [8]. However, it is not known if increasing HDL levels by CETP inhibition can ameliorate NASH.

Cholesteryl ester transfer protein (CETP) is considered a therapeutic target for increasing HDL-C [9]. Interest in CETP as a therapeutic target began as a result of the high HDL-C and low LDL-C observed in Japanese people carrying the homozygous, defective CETP gene who showed no evidence of premature atherosclerosis, even though they had hypercholesterolemia [10]. This CETP is bound mainly to HDL particles and transfers cholesterol ester from HDL to triglyceride-rich lipoproteins. CETP action results in a CE enrichment of non-HDL lipoproteins, which could contribute to atherosclerosis. Small molecule CETP inhibitors, including dalcetrapib, evacetrapib, and anacetrapib, that are at various phases of clinical development inhibit CETP activity and significantly increase HDL-C [11]. However, torcetrapib was failed in phase 3 clinical trial due to compound specific off-target effects [12], and dalcetrapib failed in phase 3 clinical trials due to less meaningful outcomes [13]. Two other CETP inhibitors, evacetrapib and anacetrapib, which showed beneficial effects by increasing HDL-C, are still under clinical trials [14].

Inducing an immune response against specific self-peptides is potentially beneficial for the treatment of certain diseases. The drawbacks of peptide-based immunization include low immunogenicity of self-peptides, a low efficiency of chemical conjugation, and the heterogeneous nature of antigen preparations. To address these problems, we have investigated the use of peptide repeats conjugated to the receptor-binding domain of Fc to induce antibodies that might suppress the function of self-proteins. Since CETP is a self-antigen with low immunogenicity, which may lead to a low immune response [15], we thus designed a novel anti-CETP vaccine (Fc-CETP_6_) composed of the Fc domain of rabbit IgG fused to a linear repeats epitope within a region responsible for CETP-VLDL/LDL binding. The Fc domain binds to the Fc receptor, facilitating antigen delivery to antigen-presenting cells through receptor-mediated endocytosis [16], [17], which is more efficient than phagocytosis, leading to antigen presentation through the MHC II pathway [18]. It has been demonstrated that carrier proteins bearing linear repeats of epitope are highly effective in inducing strong B cell activation [19]. In this study, linear array epitope (LAE) technology was applied to generate linear repeating epitope [20].

Rabbits have higher CETP levels than humans and are highly susceptible to the induction of developing atherosclerosis [21] and NASH [22]. In this study, the rabbits were fed a high fat/cholesterol (HFC) diet to induce atherosclerosis and NASH. Efficacy of the Fc-CETP_6_ vaccine in alleviating the progression of atherosclerosis and NASH was evaluated. Our results showed that the Fc-CETP_6_ vaccine successfully elicited anti-CETP antibodies and lowered plasma CETP activity. Pathological analyses revealed less lipid accumulation and inflammation in the aorta and liver of the Fc-CETP_6_-immunized rabbits.

## Materials and Methods

### Construction of DNA fragment, encoding for 6 repeats of human CETP epitope, followed by fusing with rabbit Fc and expression of the fused protein in *E. coli* BL21 (DE3)

**Figure 1:**
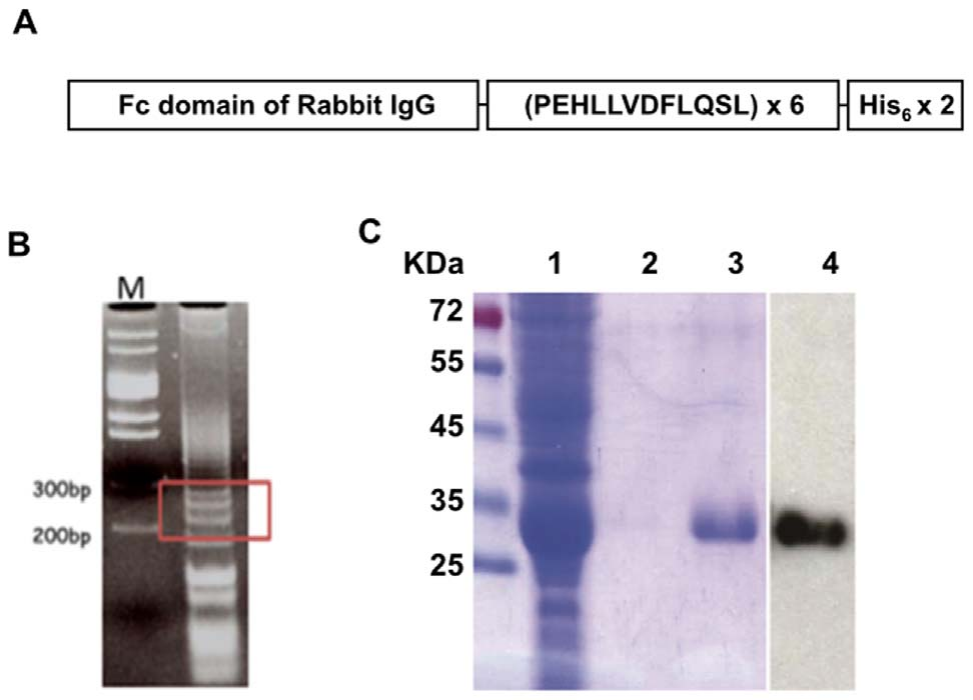
The Strategy for produces the Fc-CETP_6_ vaccine. (A) Schematic diagram of Fc-CETP_6_. The CETP vaccine contained the Fc domain of rabbit IgG, followed by 6 repeats of the human CETP epitope, and a double-His_6_-tag. (B) The products of adapter-PCR. The 200–300 bp products were boxed. M stands for DNA marker. (C) Purity of the His-Bind-purified Fc-CETP_6_ immunogen. Lanes 1 to 3 show Coomassie blue staining results for the flow-through fraction, wash fraction, and elutes, respectively, while lane 4 shows Western blot analysis of elutes using anti-rabbit IgG (Fc) antibodies.

The DNA fragment encoding for 6 repeats of the peptide PEHLLVDFLQSL, a human CETP epitope [23], was generated by the template-repeated polymerase chain reaction (TR-PCR), as described previously [20] (Fig. 1). The TR-PCR products were then subjected to adapter-PCR using adapter primers (Table S2) to create restriction sites at the 5′- and 3′-ends to facilitate further subcloning. The 200–300 bp adapter-PCR products (Fig. 1B) were eluted and cloned into the T-Easy vector (Promega). A clone containing 6 copies of the CETP epitope was identified by sequencing and subcloned into a modified plasmid pET21b vector (Novagen) at the 3′-end of the region coding for the Fc domain of the rabbit IgG. The resulting plasmid, pFc-CETP_6_, was confirmed by sequencing and introduced into *E. coli* BL21 (DE3), and the expressed fusion protein (Fc-CETP_6_) was purified by chromatography on a His-Bind column (Novagen).

### Assay of anti-CETP antibody titers

The GST and GST-CETP proteins were expressed in E.*coli* Top10 (Invitrogen), and purified by affinity chromatography. ELISA was applied using plates coated with GST-CETP or GST as tagging control to determine plasma antibody titers to CETP. The titer was defined as the plasma dilution that gave an optical density of 1.0 at 405 nm.

### Animal experiments

Two animal studies were carried out; in both, 16-week-old female New Zealand White rabbits (∼2 kg) were allowed an adaptation period of 2 weeks, and then treated as follows.

In the first experiment, the rabbits were randomly allocated to the normal group (n = 4), control group (n = 7), or Fc-CETP_6_ group (n = 8). Rabbits in the normal group were fed a regular chow diet, while rabbits in the control and Fc-CETP_6_ groups were fed the HFC diet (chow supplemented with 5% lard and 0.25% cholesterol). Each animal was fed 100 g of the diet per day during the study period and allowed free access to tap water. Rabbits in the Fc-CETP_6_ group were injected subcutaneously with 0.1 mg of Fc-CETP_6_ on day 1 and at the end of weeks 2, 4, 6, and 8 (first injection in complete Freund's adjuvant and all others in incomplete Freund's adjuvant), while the control HFC diet-fed group underwent the same schedule of injections with PBS in adjuvant. Plasma samples were collected at the end of weeks 1, 8, 12, 20, and 24 and CETP activity and titers of anti-CETP antibodies were measured. At week 24, the rabbits fasted overnight, and the next morning anesthetized by inhalation of isoflurane and the rabbits' aortas were also collected for atherosclerotic lesion analysis.

In the second experiment, the rabbits were treated as in the first experiment except that the Fc-CETP_6_ group rabbits (n = 8) received booster injections with 0.1 mg of Fc-CETP_6_ in incomplete Freund's adjuvant at the end of weeks 24 and 28 and were sacrificed at the end of week 52. After overnight fasting, the rabbits were anesthetized as above and their liver removed after perfusion with physiological saline. Then three samples (each approximately 1 cm^3^) of the right lobe were taken, fixed in 4% paraformaldehyde, and embedded in paraffin.

All procedures for the care and use of research animals were in accordance with the guidelines of, and approved by, the animal center of the Taiwan Food and Drug Administration, Department of Health, Executive Yuan.

### Detection of CETP activity

Plasma CETP activity was measured using CETP Activity Assay Kits (BioVision) according to the manufacturer's guidelines. The data are presented as a percentage of the levels before treatment.

### Isolation of lipoprotein fractions and measurement of cholesterol levels

The procedures used to isolate lipoprotein fractions have been described previously [24]. In brief, plasma was prepared by low speed centrifugation at 4°C, then VLDL, LDL, and HDL were isolated by density gradient ultracentrifugation and cholesterol concentrations in the plasma and lipoprotein fractions determined enzymatically (Randox) according to the manufacturer's guidelines.

### Quantification of atherosclerotic lesions in the aorta

The aorta was perfused for 20 min with ice-cold PBS, and then pressure-fixed with cold formaldehyde-sucrose solution (10% neutral formalin, 5% sucrose, 20 mM butylated hydroxytoluene, and 2 mM EDTA, pH 7.4). The entire aorta was then dissected out and the aorta opened longitudinally, rinsed briefly in 70% ethanol, and stained with Sudan IV (0.5% Sudan IV in 35% ethanol/50% acetone), and de-stained for 5 min with 80% ethanol. Each aorta was then mounted on a flat surface and images of its surface taken using a digital camera. The area stained with Sudan IV (lipid plaque) was expressed as a percentage of the total surface area of the aorta.

### Immunohistochemical analysis

The procedures used have been described previously [25]. In brief, levels of NF-kB and RAM-1 expression in the aorta and liver tissue were determined by immunohistochemical staining using mouse anti-NF-kB and anti-RAM-11 antibodies (Dako). The aortas were analyzed from cross section from each animal and ten random fields (magnification, x200) of each cross-section photographed under a microscope (Olympus, BX60). The liver tissues were analyzed from three tissue sections from right lobe of each animal and ten random fields (magnification, x200) of each section photographed under a microscope (Olympus, BX60).

### Liver histology and quantification of steatosis and fibrosis in the liver

Liver sections (4 µm) were stained with hematoxylin and eosin (H&E) and Masson's trichrome stain. An expert pathologist assessed histological steatosis and fibrosis stages with the Histological Scoring System for Nonalcoholic Fatty Liver Disease (NAFLD) [26]. Three tissue sections from each animal were analyzed and ten random fields (magnification, x200) of each section photographed under a microscope (Olympus, BX60).

### Measurement of plasma ApoA-I and oxidized–LDL levels

Plasma ApoA-I and plasma ox-LDL were measured using sandwich ELISA kits from CUSABIO (CSB-E09804Rb) and Mercodia (10-1158-01), respectively, according to the manufacturer's guidelines.

### Statistical analysis

All data are presented as the mean ± SEM. Normality was examined by the Kolmogorov-Smirnov test, and when the data was normally distributed, the statistical significance of differences was assessed with the independent t test and 1-way ANOVA and by correlation and regression analysis using the SPSS statistical program, version 17. The Mann–Whitney U test was applied when the data was not normally distributed. In all analyses, values of P<0.05 were considered statistically significant.

## Results

### Generation of the Fc-CETP_6_ vaccine

DNA encoding 6 repeats of CETP epitope was generated and the fusion protein was induced and expressed in *E.coli* strain BL21 (DE3) as shown in Fig. 1. The fusion protein Fc-CETP_6_ was purified by affinity chromatography on a His-Bind column. The purity of the Fc-CETP_6_ fusion protein examined by Coomassie Blue staining and Western blotting reached 95% (Fig. 1C).

### Fc-CETP_6_ vaccine elicits antibodies against CETP and reduces CETP activity in HFC diet-fed rabbits

**Figure 2:**
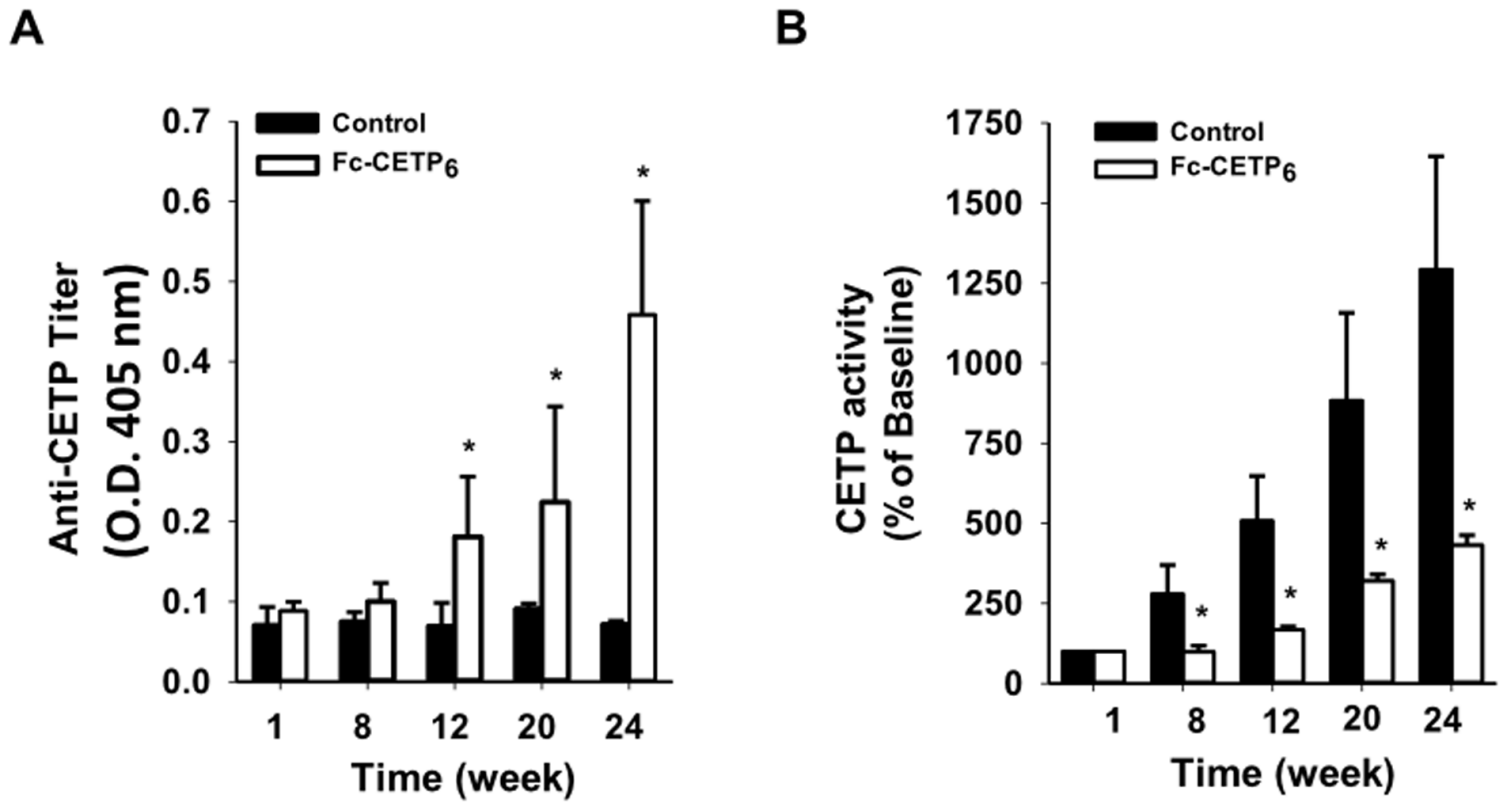
Measurement of plasma anti-CETP titers and CETP activity in rabbits immunized with Fc-CETP_6_ (experiment 1). (A) Plasma antibody titers against CETP. Plasma samples were collected as described in material and methods (Control group n = 7; Fc-CETP_6_ group n = 8) and diluted 2000-fold and the anti-CETP titer measured by ELISA. (B) Plasma CETP activity. Plasma CETP activity was measured and expressed as a percentage of that in the pre-vaccination sample (Control group n = 7; Fc-CETP_6_ group n = 8). Values are the mean ± SEM for the control rabbits fed the HFC diet and the rabbits fed the HFC diet and immunized with Fc-CETP_6_. *p<0.05 compared to the control at the same time.

To demonstrate that anti-CETP antibodies were produced and reacted with circulating CETP, the plasma anti-CETP titer and plasma CETP activity were measured. Fig. 2A shows that anti-CETP antibodies were detected in the Fc-CETP_6_ group from week 12 and that the titer increased up until the end of the study at week 24. Fig. 2B shows that plasma CETP activity in both HFC diet-fed groups increased in a time-dependent manner, but was significantly lower in the Fc-CETP_6_ group. These results show that injection of Fc-CETP_6_ induces production of antibodies against CETP, which then reduce CETP activity.

### Effects of the Fc-CETP_6_ vaccine on plasma levels of total-cholesterol and HDL-cholesterol

The effects of vaccination with Fc-CETP_6_ on levels of total-C and HDL-C and body weight are summarized in Table 1. There was no significant difference between the control and Fc-CETP_6_ groups in body weight. Levels of total-C and HDL-C increased in a time-dependent manner in both groups. At weeks 12 and 24, total-C levels were significantly lower, and HDL-C levels significantly higher, in the Fc-CETP_6_ group than in the control group. In addition, at weeks 12 and 24, the non-HDL-C/HDL-C ratio was lower in the Fc-CETP_6_ group than in the control group. These results show that Fc-CETP_6_ immunization resulted in a reduced atherogenic lipoprotein profile.

**Table 1:** Changes in body weight and plasma total cholesterol and HDL-C in rabbits fed the HFC diet with or without vaccination.

Group	Time (week)	Body wt (kg)	Total C (mg dl^−1^)	HDL-C (mg dl^−1^)	Non-HDL-C/HDL-C
Control	0	2.6±0.1^1^	127.0±2.7	24.3±3.7	4.5±0.9
	12	3.3±0.3	342.7±44.5	40.4±5.4	7.5±0.2
	24	3.9±0.3	454.6±10.9	46.6±7.2	8.8±2.4
Fc-CETP_6_	0	2.5±0.2	159.0±19.9	29.8±5.0	4.9±1.0
	12	3.2±0.3	262.0±18.8^*^	52.8±10.7^*^	4.7±0.9^*^

Control n = 7; Fc-CETP6 n = 8. ^1^Values are the mean ± SEM. *p<0.05 compared to the control at the same time.

### Fc-CETP_6_ increases ApoA-I levels and lowers ox-LDL levels

ApoA-I is a major protein in HDL and is known to have anti-inflammatory effects [27]; high levels of ApoA-I have been shown to reduce the progression and even induce regression of atherosclerosis, indicating that ApoA-I is directly protective against atherosclerosis [28]. In addition, ox-LDL is taken up by macrophages in the artery and transforms them into cholesterol-rich foam cells that have been shown to involved in the development of NASH [29]. We therefore measured plasma levels of ApoA-I and ox-LDL in the rabbits (Table 2) and found that ApoA-I levels were significantly higher, and ox-LDL levels significantly lower, in the Fc-CETP_6_ group than in the control group. In addition, the immunofluorescence stain showed the expression level of ox-LDL around the vein area of liver was significantly reduced in the Fc-CETP_6_ group compared to control group (Figures S1 and S2). These results may help explain why vaccination with Fc-CETP_6_ alleviated development of HFC diet-induced atherosclerosis and NASH.

**Table 2:** Plasma levels of ApoA-I and ox-LDL.

Plasma levels	Control	Fc-CETP_6_
ApoA-I (µg mL^−1^)	22.2±7.5^1^	96.2±30.6^*^
ox-LDL (unit L^−1^)	61.6±1.8	49.7±5.2^*^

Control n = 7; Fc-CETP6 n = 8. ^1^Values are the mean ± SEM. *p<0.05 compared to control.

### Fc-CETP_6_ ameliorates HFC diet-induced atherosclerotic lesions and inflammation in the aorta

**Figure 3:**
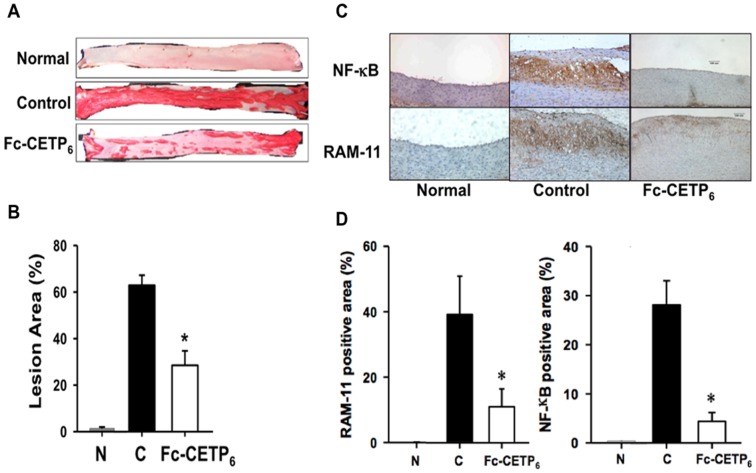
Vaccination with Fc-CETP_6_ reduces Aortic lesions and inflammation (experiment 1). (A) Representative Sudan IV-stained aortic specimens at the end of week 24. (B) Quantification of the aortic lesion area. The bar graphs represent the average (N: normal group n = 4; C: control group n = 7; Fc-CETP_6_ group n = 8) for each group with standard errors. *p<0.05 compared to the control. (C) Expression of NF-κB and RAM-11 (magnification, x200) of the aorta from normal rabbits, control rabbits, and Fc-CETP_6_ rabbits. (D) Quantification of the RAM-11 and NF-κB positive area of aorta. The bar graphs represent the average (N: normal group n = 4; C: control group n = 7; Fc-CETP_6_ group n = 8) for each group with standard errors. The positive-stained area was calculated as the (stained area/total area) x 100. Values are the mean ± SEM. *p<0.05 compared to the control.

To examine whether vaccination with Fc-CETP_6_ could ameliorate formation of atherosclerotic plaques, the aorta from each rabbit was isolated and stained with Sudan IV. Representative Sudan IV-stained aorta from each group is shown in Fig. 3A, and the extent of atherosclerosis in the entire aorta, quantified using an image analysis system, is shown in Fig. 3B. In the normal rabbits, only about 1% of the aorta surface area stained by Sudan IV. In contrast, rabbits maintained on the HFC diet for 24 weeks developed atherosclerosis lesions that covered 62.9±4.3% of the aortic surface in the control group, but only 28.5±6.2% in the Fc-CETP_6_ group (Fig. 3B). Since atherogenesis involves long-term inflammation and the macrophage is considered a key mediator in aortic local inflammation [30], an immunohistochemical analysis was performed to detect the presence of NF-kB, an inflammatory marker, and RAM-11, a macrophage marker, in the aorta. Fig. 3C shows that no NF-kB or RAM-11 staining was detected in the aorta of the normal rabbits, whereas the HFC diet resulted in strong RAM-11 and NF-kB staining in the intima in the control group. Both effects were significantly reduced by vaccination with Fc-CETP_6_ (Fig. 3D). In summary, our results show that immunization with Fc-CETP_6_ reduced the extent of aortic inflammation and macrophage infiltration, and thus decreased susceptibility to atherosclerosis progression in the rabbit aorta.

### Fc-CETP_6_ alleviates HFC diet-induced NASH and fibrosis

**Figure 4:**
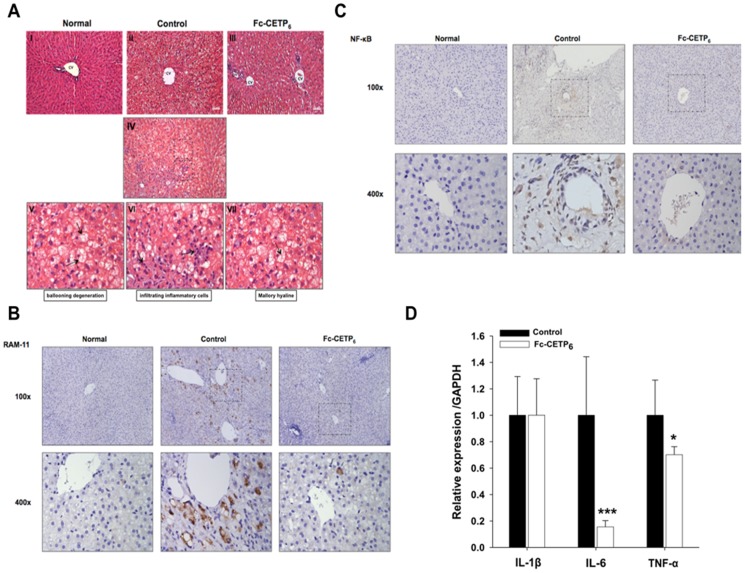
Vaccination with Fc-CETP_6_ attenuates high HFC diet-induced steatosis and NASH. (A) Representative liver sections stained with H&E (magnification, x200). Liver section stained with H&E from control rabbits showing NASH characteristics, such as ballooning degeneration, infiltrating inflammatory cells and Mallory hyaline were observed in the control (magnification, x200). (B) Expression of RAM-11 (magnification, upper panel x100 and lower panel x400) of the liver from control and Fc-CETP_6_ rabbits. (C) Expression of NF-kB (magnification, upper panel x100 and lower panel x400) of the liver from control and Fc-CETP_6_ rabbits. (D) Expression of inflammation related genes in control rabbits (black bars) and Fc-CETP_6_ rabbits (white bars). The bar graphs represent the average (C: control group n = 7; Fc-CETP_6_ group n = 8) for each group with standard errors. Values are the mean ± SEM. *p<0.05; ***p<0.001 compared to the control.

NASH is characterized by hepatic lipid accumulation combined with inflammation, which may ultimately progress into cirrhosis. In the first experiment designed to examine whether the vaccine was able to alleviate the atherosclerosis induced by the HFC diet, we observed that the livers from the control group were pale in color, while those from the normal group and Fc-CETP_6_ group were red, suggesting that Fc-CETP_6_ injection alleviated HFC-induced nonalcoholic fatty liver disease. Ogawa et al. found that a human-type NASH with advanced fibrosis is induced in rabbits by feeding a diet supplemented with 0.75% cholesterol and 12% corn oil [22]. In the second experiment, to test whether the Fc-CETP_6_ vaccine could reduce susceptibility to NASH and fibrosis in rabbits fed a HFC diet, we examined the effects of the Fc-CETP_6_ vaccine on hepatic lipid accumulation and NASH in rabbits after 12 months of HFC diet feeding by staining of liver sections with H&E and Masson's trichrome. As shown in Fig. 4, liver sections from the control group showed evidence of parenchymatous lipid accumulation, microvesicular steatosis with ballooning degeneration in the perivenular area, infiltrating inflammatory cells, and Mallory hyaline (Fig. 4A V-VII), whereas those from the Fc-CETP_6_ group showed hepatocytes with only mild fat accumulation, and those from normal group showed no evidence of steatosis. To further evaluate the effects of Fc-CETP_6_ on liver inflammation, immunohistochemistry staining were performed using antibodies against RAM-11, recognizes activated kupffer cells [31], and NF-κB, an inflammation marker. Compared to the liver of control rabbit, the liver of Fc-CETP_6_ showed decreased number of RAM-11 positive cell around the central vein area at sites where fatty degeneration of hepatocytes was evident. Normal group was shown as RAM-11 negative control (Fig. 4B). At higher magnification, large RAM-11 positive cells contained vacuoles (lipid droplet). Moreover, NF-κB stained cells around central vein area in the liver of control rabbits, and NF-κB stained cells were significantly reduced in the Fc-CETP_6_ group. Normal group was shown as NF-κB negative control (Fig. 4C). The changes of inflammation and fibrosis were further confirmed by detection of hepatic mRNA expression levels of pro-inflammatory cytokines and genes associated with fibrosis. Fig. 4D shows that mRNA levels of tumor necrosis factor alpha (TNFα) and interleukin-6 (IL-6) were significantly lower in the Fc-CETP_6_ group than in the control group; whereas (interleukin 1β) IL-1β was not different between the two groups. These results support that Fc-CETP_6_ reduces hepatic inflammation in the HFC diet fed rabbits.

**Figure 5:**
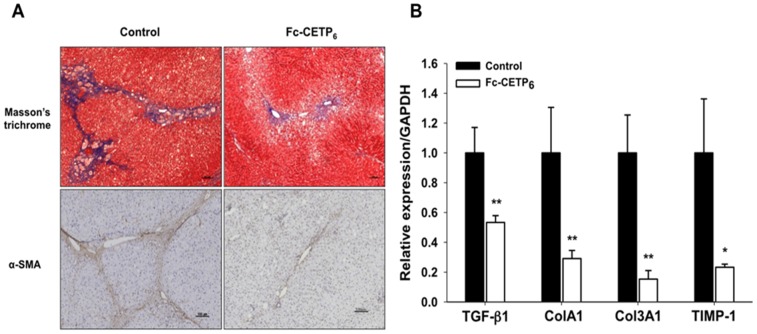
Vaccination with Fc-CETP_6_ reduces inflammation related gene and fibrosis related gene expression profile (experiment 2). (A) Representative liver sections stained with Masson's trichrome stain and immunohistochemistry stained for α–SMA, a marker of activated stellate cells and myofibroblasts. α–SMA positive cells were expressed near the fibrotic septa (magnification, upper panel x100 and lower panel x100). Three sections from right lobe liver of each rabbit (control group n = 7; Fc-CETP_6_ group n = 8) were analyzed and ten random fields (magnification, x200) of each section photographed under a microscope (Olympus, BX60). (B) Expression of fibrosis related genes in control rabbits (black bars) and Fc-CETP_6_ rabbits (white bars) *p<0.05; **p<0.01compared to the control.

Liver sections from the control group showed positive trichrome staining, prominent bridging fibrosis with fibrous bands extending from the perivenular area to the pericellular area, whereas the Fc-CETP_6_ group showed diminished, or no, bridging fibrosis. The fibrotic septa were composed of cells positive for α-SMA, a marker of activated stellate cells and myofibroblasts (Fig. 5A); whereas the Fc-CETP_6_ group showed diminished bridging fibrosis that limited in central vein area. And hepatic mRNA levels of fibrosis associate genes, transforming growth factor β1 (TGF-β1), collagens 1A1 (Col1A1), 3A1 (Col3A1) and tissue inhibitors of metalloproteinases-1 (TIMP-1) were all significantly lower in the liver of Fc-CETP_6_ rabbits than in the liver of control rabbits (Fig. 5B). These results support that Fc-CETP_6_ reduces hepatic fibrosis in the HFC diet fed rabbits.

The grades of steatosis and fibrosis were summarized in the Table S1. In terms of steatosis, the results showed that, of the liver specimens from the control group, none were grade 0 or 1, 7% were grade 2, and 93% grade 3, whereas the values for the Fc-CETP_6_ group were 12% grade 0, 56% grade 1, 32% grade 2, and none grade 3. In terms of fibrosis, in the control group, none were grade 0 or 1, while 25% were grade 2, 62.5% grade 3, and 12.5% grade 4, whereas in the Fc-CETP_6_ group, 14% of the liver specimens presenting no fibrosis, while 29% were grade 1a, 43% grade 1b, 14% grade 2, and none grade 3 or 4 (Table S1).

## Discussion

In this study, we designed and produced a novel Fc-CETP_6_ vaccine. This vaccine induced the generation of anti-CETP antibodies that reduced plasma CETP activity, alleviating the development of both atherosclerosis and NASH in HFC diet-fed rabbits. Rittershaus et al. showed that a decrease in CETP activity *in vivo* by vaccination with TT/CETP vaccine could enhance HDL-C and reduced atherosclerosis [32]. To our knowledge, our study provides the first evidence showing that a vaccine targeted at CETP is able to delay the development of both atherosclerosis and NASH in HFC diet-fed rabbits. Atherosclerosis and NASH are both metabolic syndrome and there is evidence that both are associated with lower HDL-C levels and higher ox-LDL levels [29] and involve macrophage activation and infiltration, accompanied by chronic inflammation [33].

In this study, we observed that the Fc-CETP_6_ vaccine could increase circulating levels of HDL-C and ApoA-I, and decrease levels of non-HDL-C and ox-LDL. Furthermore, Fc-CETP_6_ vaccine can reduce macrophage infiltration (RAM-11) and activation (NF-kB) in the aorta and liver tissue. Patients with hepatic steatosis also showed higher CETP activity [3], indicating that CETP inhibition may be regarded as a target to improve NASH [34]. In addition, ApoA-I, the most abundant protein in HDL, has been demonstrated to be a potent anti-oxidative and anti-inflammatory agent in *in vivo* studies [35], [36]. In this study, plasma ApoA-I levels were 4.5 times higher in the Fc-CETP_6_ group than in the control group and these high levels of ApoA-I may result in increased anti-oxidative and anti-inflammatory effects. It is not fully revealed whether CETP inhibition improves the progression of NASH and atherosclerosis, but it is worth further investigation.

Since an increased CETP level can reduce HDL-C and CETP deficiency is associated with elevated HDL-C, CETP inhibitors have been investigated. However, clinical trial results showed that the CETP inhibitors torcetrapib and anacetrapib significantly increase HDL-C levels, but do not reduce the risk of recurrent cardiovascular events [37], [38]. Several lines of evidence indicate that the increase in HDL levels caused by torcetrapib is due to a reduction in the HDL-ApoA-I catabolic rate, rather than increased HDL production [39], [40]. Unlike small molecule inhibitors [41], antibodies are non-permeable large molecules and only work at the plasma level. When the anti-CETP antibody binds to C-terminus of CETP, this process blocks the transfer of cholesterol ester from HDL to VLDL and LDL [42].

Higher CETP activity has been shown to be closely associated with hepatic steatosis in patients with metabolic syndrome [4]. Moreover, over-expression of simian CETP in 57BL/6 mice accelerates the development of fatty liver [43]. In this study, we showed that inhibition of CETP by the Fc-CETP_6_ vaccine markedly reduced the occurrence of HFC diet-induced hepatic steatosis and steatohepatitis in rabbits. Recent reports have shown that the pathogenesis of NASH involves scavenger receptor-mediated uptake of ox-LDL by macrophages in the liver [6], [7], and that NASH is strongly associated with risk of cardiovascular disease [8], [44]. It is highly likely that atherosclerosis and NASH result from the same etiology and that increasing HDL levels and reducing ox-LDL accumulation in macrophage may be beneficial in both diseases. However, the underlying mechanisms by which inhibition of CETP prevents hepatic steatosis and steatohepatitis deserve further investigation.

Based on these findings, linear array epitope with Fc as a vaccine was proven to amplify immunogenicity, even in our adjuvant-free trail (data not shown). Our Fc-CETP_6_ vaccine may have potential application in the development of a therapeutic agent for both atherosclerosis and NASH in humans. However, the underlying cause of NASH is still not clear. Obesity, diabetes, insulin resistance, or intestinal bacteria have been proposed as risk factors for its development [45]. In this study, the HFC diet-fed rabbits did not develop obesity and diabetes. It is, therefore, not clear whether an anti-CETP vaccine can prevent NASH induced by other different factors in addition to the HFC diet, but this approach definitely merits further investigation.

## Supporting Information

Figure S1 **Immunofluorescence staining of ox-LDL in liver specimens.** (A) Representative immunofluorescence staining of ox-LDL in liver specimens at the end of week 52nd. Control n = 7, Fc-CETP_6_ n = 8. Specimens stained with DAPI to visualize the nuclei (magnification, x100).
(TIF)

Figure S2 **Quantification of liver ox-LDL.** Quantification of ox-LDL positive stain area. The relative intensity in Y-axis of figure was calculated as “Mean Intensity/image captured area(µm xµm)”x100. Values are the mean ± SEM. **p<0.01.
(TIF)

Table S1 **Effects of the Fc-CETP6 vaccine on Hepatic Histology at 52 Weeks.** Vaccination with Fc-CETP_6_ attenuates HFC diet-induced NASH and Fibrosis on Hepatic Histology at the end of week 52nd. Three sections (each approximately 1 cm^3^) were obtained from the right lobe liver of each rabbit (control group n = 7, Fc-CETP_6_ group n = 8). Ten images were taken randomly from each section. Randomly select 200 samples out of each group for data quantification.
(TIF)

Table S2 **Primer list of TR-PCR, AD-PCR and Quantitative-PCR.**
(TIF)

Materials and Methods S1 Method S1. Immunofluorescence staining of the liver tissue. The presence of ox-LDL in the liver tissue sections was evaluated by immunofluorescence staining. The right lobe of each rabbit liver was obtained and embedded in OCT compound then stored at −20°C. The slides were cut to a thickness of 6 µm and fixed with ice-cold acetone. Primary antibody in 30 µl (rabbit polyclonal anti ox-LDL immunoglobulin (IgG, Calbiochem, Germany) was added. Slides incubated in the absence of primary antibody were used as negative control. After incubating for 30 min in a humid chamber at room temperature, slides were washed with PBS, and a fluorescein isothiocyanate labeled antihuman IgG (30 µl) was administered as a conjugate substance. After another 30 min at room temperature, the slides were washed with the standard PBS solution. After drying, the slides were covered with a mounting medium and examined under a fluorescence microscope (Leica DMRX, Wetzlar, Germany). **Method S2. Quantification of ox-LDL staining procedure.** The images were obtained from three sections of the right lobe liver from each rabbit (control group n = 7, Fc-CETP_6_ group n = 8). Ten images were taken randomly from each section. The relative intensity of ox-LDL positive staining was calculated from the program, Zen2010 (Carl Zeiss MicroImaging, Inc., Thornwood, NY, USA). The relative intensity in Y-axis of figure was calculated as “Mean Intensity/image captured area (µm xµm)” x100.
(DOC)
