# Supplementary figures and images for: A Vaccine Targeted at CETP Alleviates High Fat and High Cholesterol Diet-Induced Atherosclerosis and Non-Alcoholic Steatohepatitis in Rabbit
Source: PLoS One. 2014 Dec 8;9(12):e111529. doi: 10.1371/journal.pone.0111529 (PMC4259298; doi:10.1371/journal.pone.0111529)

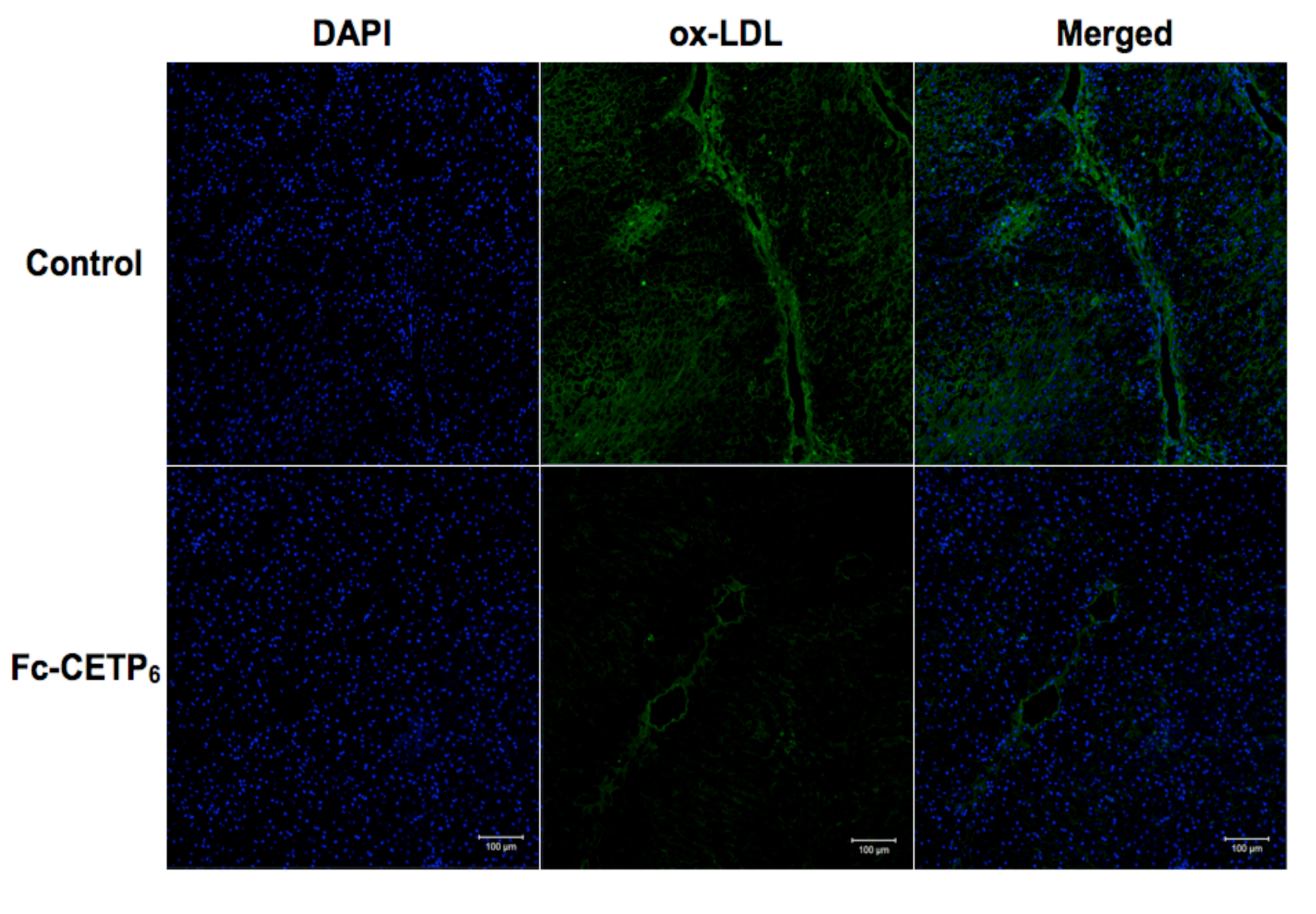

Supplement: Figure S1 — Immunofluorescence staining of ox-LDL in liver specimens. (A) Representative immunofluorescence staining of ox-LDL in liver specimens at the end of week 52nd. Control n = 7, Fc-CETP6 n = 8. Specimens stained with DAPI to visualize the nuclei (magnification, x100). (TIF) [file pone.0111529.s001.tif]

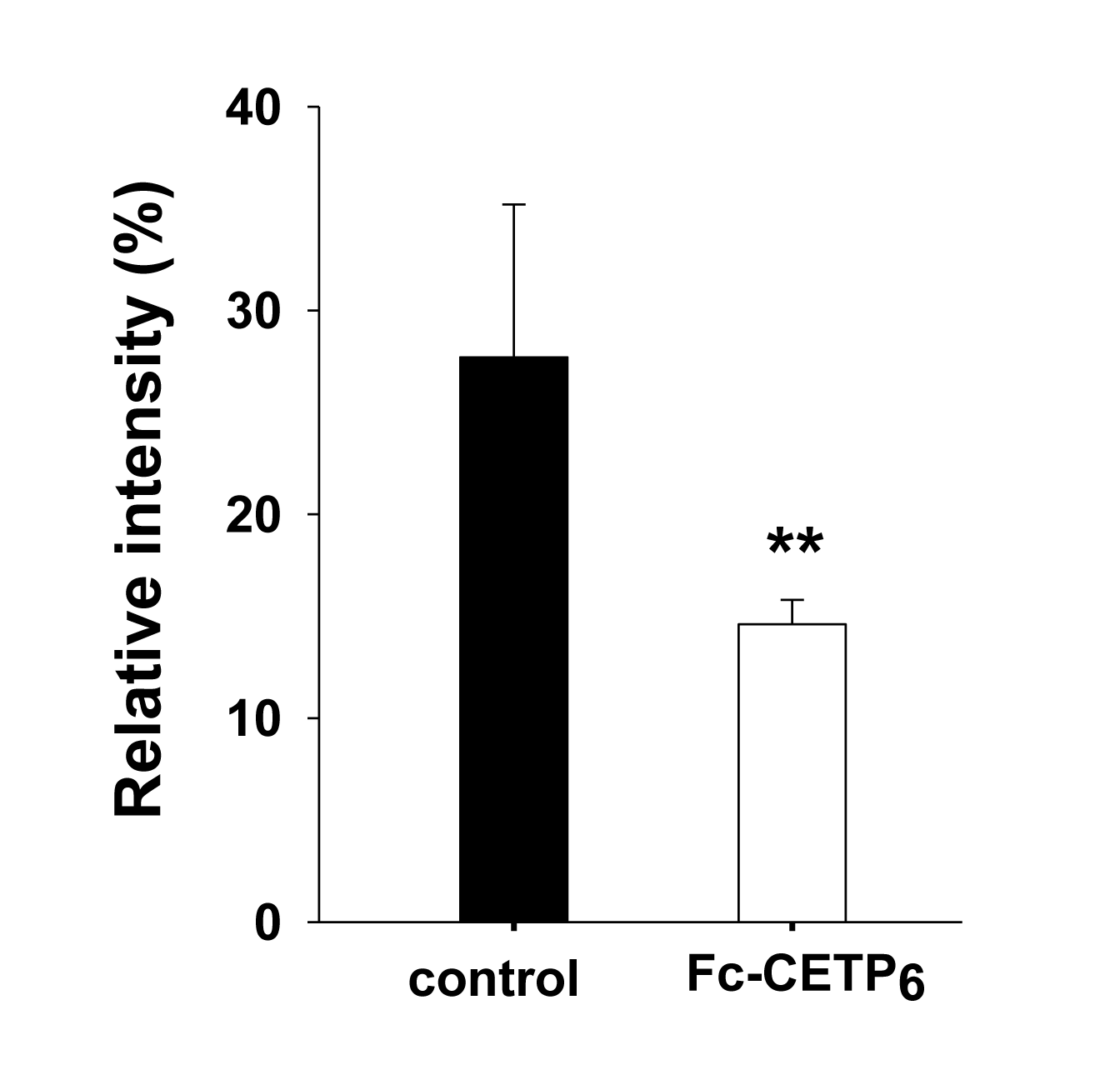

Supplement: Figure S2 — Quantification of liver ox-LDL. Quantification of ox-LDL positive stain area. The relative intensity in Y-axis of figure was calculated as “Mean Intensity/image captured area(µm xµm)”x100. Values are the mean ± SEM. **p<0.01. (TIF) [file pone.0111529.s002.tif]

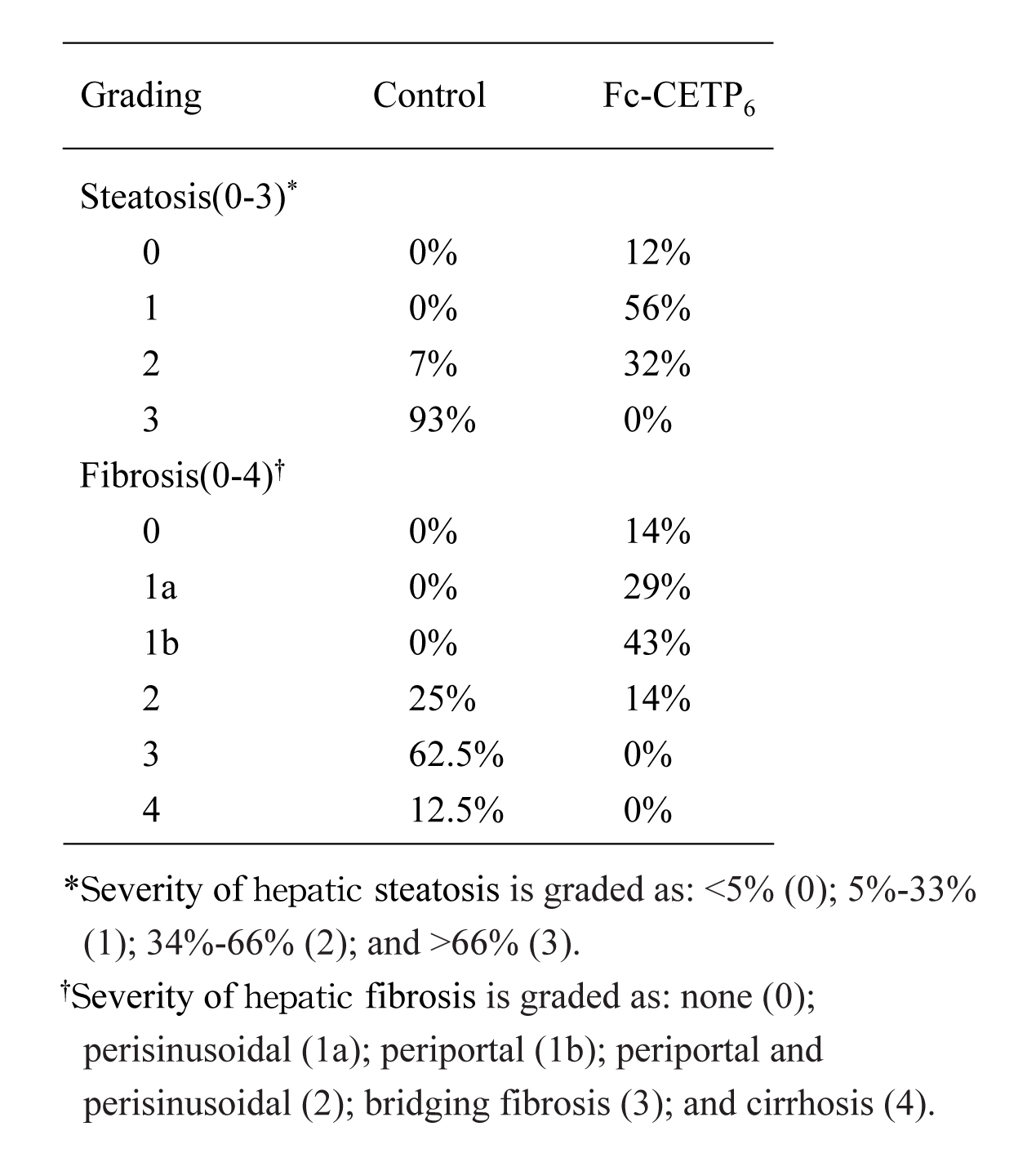

Supplement: Table S1 — Effects of the Fc-CETP6 vaccine on Hepatic Histology at 52 Weeks. Vaccination with Fc-CETP6 attenuates HFC diet-induced NASH and Fibrosis on Hepatic Histology at the end of week 52nd. Three sections (each approximately 1 cm3) were obtained from the right lobe liver of each rabbit (control group n = 7, Fc-CETP6 group n = 8). Ten images were taken randomly from each section. Randomly select 200 samples out of each group for data quantification. (TIF) [file pone.0111529.s003.tif]

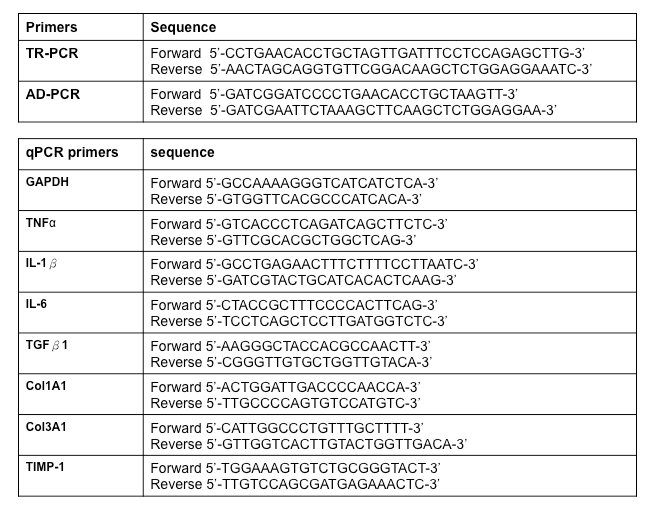

Supplement: Table S2 — Primer list of TR-PCR, AD-PCR and Quantitative-PCR. (TIF) [file pone.0111529.s004.tif]
