# Supplementary material for: A Vaccine Targeted at CETP Alleviates High Fat and High Cholesterol Diet-Induced Atherosclerosis and Non-Alcoholic Steatohepatitis in Rabbit
Source: PLoS One. 2014 Dec 8;9(12):e111529. doi: 10.1371/journal.pone.0111529 (PMC4259298; doi:10.1371/journal.pone.0111529)
Supplement: Materials and Methods S1 — Method S1. Immunofluorescence staining of the liver tissue. The presence of ox-LDL in the liver tissue sections was evaluated by immunofluorescence staining. The right lobe of each rabbit liver was obtained and embedded in OCT compound then stored at −20°C. The slides were cut to a thickness of 6 µm and fixed with ice-cold acetone. Primary antibody in 30 µl (rabbit polyclonal anti ox-LDL immunoglobulin (IgG, Calbiochem, Germany) was added. Slides incubated in the absence of primary antibody were used as negative control. After incubating for 30 min in a humid chamber at room temperature, slides were washed with PBS, and a fluorescein isothiocyanate labeled antihuman IgG (30 µl) was administered as a conjugate substance. After another 30 min at room temperature, the slides were washed with the standard PBS solution. After drying, the slides were covered with a mounting medium and examined under a fluorescence microscope (Leica DMRX, Wetzlar, Germany). Method S2. Quantification of ox-LDL staining procedure. The images were obtained from three sections of the right lobe liver from each rabbit (control group n = 7, Fc-CETP6 group n = 8). Ten images were taken randomly from each section. The relative intensity of ox-LDL positive staining was calculated from the program, Zen2010 (Carl Zeiss MicroImaging, Inc., Thornwood, NY, USA). The relative intensity in Y-axis of figure was calculated as “Mean Intensity/image captured area (µm xµm)” x100. (DOC) [file pone.0111529.s005.doc]

**S1 Materials and Methods**

**Method S1. Immunofluorescence staining of the liver tissue.** The presence of ox-LDL in the liver tissue sections was evaluated by immunofluorescence staining. The right lobe of each rabbit liver was obtained and embedded in OCT compound then stored at -20°C. The slides were cut to a thickness of 6 µm and fixed with ice-cold acetone. Primary antibody in 30 µl (rabbit polyclonal anti ox-LDL immunoglobulin (IgG, Calbiochem, Germany) was added. Slides incubated in the absence of primary antibody were used as negative control. After incubating for 30 min in a humid chamber at room temperature, slides were washed with PBS, and a fluorescein isothiocyanate labeled antihuman IgG (30 µl) was administered as a conjugate substance. After another 30 min at room temperature, the slides were washed with the standard PBS solution. After drying, the slides were covered with a mounting medium and examined under a fluorescence microscope (Leica DMRX, Wetzlar, Germany).

**Method S2. Quantification of ox-LDL staining procedure.** The images were obtained from three sections of the right lobe liver from each rabbit (control group n=7, Fc-CETP_6_ group n=8). Ten images were taken randomly from each section. The relative intensity of ox-LDL positive staining was calculated from the program, Zen2010 (Carl Zeiss MicroImaging, Inc., Thornwood, NY, USA). The relative intensity in Y-axis of figure was calculated as “Mean Intensity/ image captured area (μm xμm)” x100.
